# Massive bilocular spinal cord intramedullary lipoma of the thoracic spine

**DOI:** 10.1259/bjrcr.20170009

**Published:** 2017-06-14

**Authors:** Aikaterini Solomou, Vasileios Panagiotopoulos, Pantelis Kraniotis, Katerina Apostolopoulou, Fotis Tzortzidis

**Affiliations:** ^1^Department of Radiology, University Hospital of Patras, Patras, Greece; ^2^Department of Neurosurgery, University Hospital of Patras, Patras, Greece

## Abstract

Spinal cord intramedullary lipomas are rare, comprising 2% of intramedullary tumours. They are more often associated with spinal dysraphism, while lipomas not associated with spinal dysraphism are even less frequent, accounting for 1% of cases. The pathogenesis of spinal cord intramedullary lipomas remains unclear. MRI is the gold standard for the evaluation of these lesions. We hereby present a case of a 37-year-old male, who underwent MRI due to spastic paraparesis. MRI revealed a bilocular, spinal cord intramedullary lesion at the level of *T*_2_–*T*_5_, with dilatation of the spinal canal and signal characteristics compatible with lipoma. There was no clear imaging evidence of spinal dysraphism. The patient underwent surgery and diagnosis was confirmed histopathologically.

## Clinical presentation

A 37-year-old male presented with a 5-year history of intermittent thoracic back pain and gradually worsening gait disturbance, associated with pain and numbness, mainly in his right lower extremity. The patient had never before consulted a doctor for his symptoms and therefore no previous imaging studies were available. He had no significant past medical history.

A complete neurological examination revealed spastic paraparesis, with muscle weakness, brisk reflexes and extensor plantar reflexes (Babinski sign) in both lower limbs. Furthermore, there was proprioceptive sensation disturbances and myoclonus of the right lower limb.

## Investigations/imaging findings

**Figure 1.**
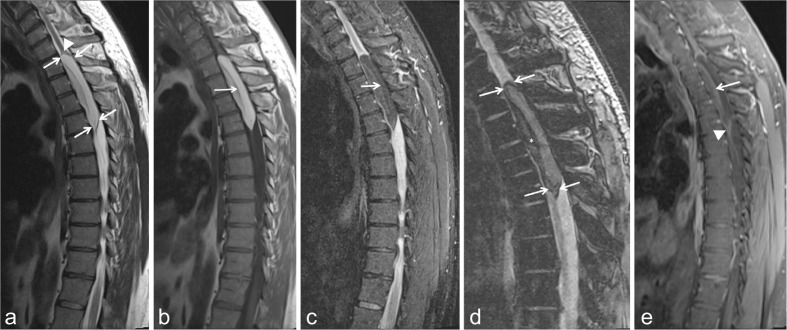
(a) Sagittal TSE *T*_2_ weighted image. There is a hyperintense intramedullary fusiform, bilocular lesion. The lesion spans along four vertebral bodies at the level of *T*_2_–*T*_5_ (arrows). There is splaying of the medulla superiorly (arrowhead). The two compartments of the lesion are separated by a low signal line. (b) Sagittal *T*_1_ weighted image. The lesion demonstrates high signal intensity, consistent with fat content. The septum between anterior and posterior compartment is hypointense (arrow). (c) Sagittal STIR. Both compartments exhibit low signal, confirming fat content. The linear septum between the anterior and posterior compartment is hyperintense (arrow). (d) Sagittal GRE. On the SWI, the margins of the lesion are low signal, probably due to chemical shift artifact (arrows). There is some degree of blooming (asterisk) in the anterior compartment, which could represent either hemosiderin deposits or microcalcifications. (e) Sagittal post Gad *T*_1_ weighted image with fat suppression. There seems to be a faint enhancement of the septum (arrow), between the two components and some degree of peripheral linear enhancement around the anterior component of the lesion (arrowhead).

The patient was submitted to MRI of the thoracic spine. *T*_1_, *T*_2_ weighted images with and without fat suppression were obtained in the axial, sagittal and coronal planes, pre- and post-IV gadolinium administration. The MRI revealed a spinal cord intramedullary lesion at the level of *T*_2_–*T*_5_ measuring 8.5 × 2.1 × 1.5 cm, causing dilatation of the spinal canal. The subarachnoid and epidural spaces were obliterated. The lesion was bilocular, consisting of anterior and posterior compartments, separated by a fine septum. On *T*_1_ and *T*_2_ weighted images the lesion was hyperintense (Figure 1a,b). On fat suppression both compartments were hypointense (Figure 1c). Minor blooming was evident on susceptibility weighted images, most likely due to hemosiderin deposits (Figure 1d). There was no evidence of nodular enhancement within the lesion after IV contrast administration, except of a faint, incomplete, linear peripheral enhancement, extending to the septum, separating the anterior and posterior part of the lesion (Figure 1e). The findings were compatible with intramedullary lipoma. The differential diagnosis would include:

**Figure 2.**
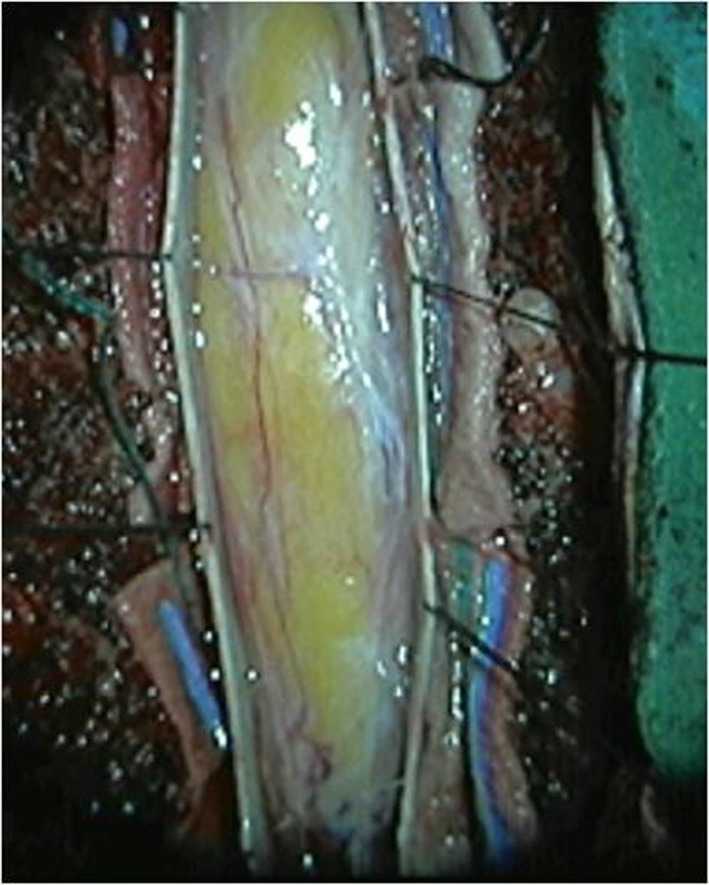
Intraoperative image, showing a fatty mass underneath the dura.

(a) Lipomyelomeningocele, which is a type of occult spinal dysrapism. A palpable mass may be found in the region. Cutaneous stigmata may coexist. There was no evidence of meningocele in our case. (b) Dermoid cyst which tends to have more heterogeneous signal intensity, and is hypointense on *T*_1_ weighted images. It may be associated with a dermal sinus. In our case the signal was homogeneously suppressed on STIR images, making the diagnosis of dermoid unlikely.

On the MRI there was no clear evidence of associated spinal fusion abnormalities.

## Treatment/outcome

The patient was submitted to surgery, under intraoperative radiography and neurophysiological monitoring with electromyography and evoked potentials. A dorsal midline incision was made, in the prone position, in order to perform a *T*_2_ to *T*_5_ laminectomy, opening of the dura and subtotal removal of the lesion. The intraoperative appearance of the lesion is seen in figure 2. There was no evidence of dysraphism. Postoperatively, the patient had a stable neurological status, compared to his pre-operative status. The histopathology report of the excised tissue mentioned the presence of fragments of mature adipose tissue.

## Discussion

Spinal cord intramedullary lipomas are rare lesions accounting for 1% of all spinal masses and 2% of intramedullary tumours.^1^ Most of them are associated with spinal dysraphism such as spina bifida and lipomeningocele. Conversely spinal cord intramedullary lipomas without spinal dysraphism are extremely rare, accounting for 1% of cases. There was no evidence of spinal dysraphism in our case. In 1876, Gowers was the first to describe an intraspinal lipoma.^2,3^

Spinal cord intramedullary lipomas are more often in young people, especially in the second and third decades of life. Males and females are equally affected. The classical location of these tumours is intradural and they may be intramedullary, extramedullary or a combination of the two.^4^

These lesions are likely to develop at the cervicothoracic, thoracic spine or cauda equina region, but may also involve the entire length of the cord and extend to the foramen magnum.^5^ In children they are more often in the cervical spine.

Histologically, spinal cord intramedullary lipomas consist of mature fat cells, with intervening collagen fibres; admixed nerve bundles are often located at the periphery, suggestive of secondary entrapment of adjacent nerve roots. Many theories have been proposed for the origin of these lesions. “Developmental error theory” is the most accepted. According to this theory, embryologic inclusion of misplaced adipocytes during the formation of the neural tube closure gives rise to lipomas and explain the dorsal location of these lesions as well as that some spinal lipomas are unassociated with dysraphism.^1,6^ “Metaplasia theory” is the second hypothesis according to which connective tissue metaplasia contribute to fat deposition within the dura.^7,8^ “Hamartomatous origin theory” is the third one, where fat tissue can include peripheral nerve twig, skeletal muscles, dermoid cyst and lymphoid or renal tissue, which originate from ectoderm or mesoderm.^7^ The fourth hypothesis points out that adipocytes may derive from cells giving rise to spinal vessels. Normally, mesenchymal cells form the spinal vessels and these cells are prevented from forming adipocytes by neural crest cells. However, if neural crest cells are abnormal, the inhibition fails and mesenchymal cells form adipocytes.^9^

Patients with spinal cord intramedullary lipomas usually present with a slow, indolent, progressive deterioration of neurologic function with hypotonia, leg weakness, loss of sense position and gait disturbance.^3^

Ascending spastic paralysis of one or more limbs is the most frequent presenting symptom. Sensory disturbances in the form of non-radiating pain, urinary incontinence, ataxia and signs of dorsal column impairment can be present and may exceed the motor symptoms.

Rapid progression of lipomas leads to neurological dysfunction, in cases of lipomas extending to the whole spinal canal.^7^ Lipomas located to the cervical spine may show protracted symptoms.^10^ If the lesion is located in the posterior aspect of the spinal canal patients may present with dorsal column dysfunction, ataxia and numbness of extremities.

MRI is considered the gold standard for the identification and the differential diagnosis of lipomas. Additionally, due to its multiplanar imaging capability, it can provide valuable preoperative information. Not only does MRI confirm the fat component of the lesion, but it also delineates its relationship with the adjacent structures. Lipomas show high signal intensity on *T*_1_ and *T*_2_ weighted images due to short *T*_1_ relaxation time of fat and its long *T*_2_ relaxation time respectively. Fat-suppressed images are valuable, confirming the presence of fat. Post-gadolinium images do not reveal internal enhancement of the lesion. In our case the faint peripheral linear enhancement may be presumably attributed to either the presence of a thin peripheral capsule or pseudocapsule of the lesion.

In cases where MRI is contraindicated, CT may be performed. CT imaging will reveal a fat density intramedullary mass. The spinal cord will have expansible appearance in the region.

Treatment of intramedullary lipoma is controversial and depends on symptoms. In asymptomatic patients no treatment is required, while in symptomatic patients surgical decompression is sufficient.^1^

Early surgical decompression prevents irreversible spinal cord dysfunction, because most symptomatic patients usually do not substantially improve after surgery.^10–12^ Therefore, the surgical planning should be decompression before symptom progression. These lesions are benign; however complete surgical removal is virtually impossible due to their vast attachment to the surrounding neural tissues. In our case, due to the infiltrative nature of the lesion and extensive adhesions of the lipoma, complete removal was also impossible. Moreover, it should be noted that aggressive surgical removal of lipomas may lead to parenchymal damage and impaired neurological functions. Recent technical advances, like the ultrasonic aspirator, surgical laser and operating microscope have significantly improved surgical management of patients with spinal cord intramedullary lipomas. The ultrasonic aspirator can be used for debulking intramedullary lipomas; however it can still be dangerous for the spinal cord.^12^ Laser surgery may be useful for gentle debulking of these tumours.^12^

Prognosis depends on the extent of the lesion and the extent of neurological dysfunction. Early diagnosis and treatment provides a better outcome. Concerning symptomatic patients, 40% show motor improvement after surgery.^13^ Patients with severe neurological symptoms have a very poor prognosis with no improvement after surgical resection.

In the English literature there are no reports concerning bilocular spinal cord intramedullary lipomas, to our knowledge. Massive spinal cord intramedullary lipomas (i.e. measuring >5cm in length) are uncommon, and only few are reported in the literature.^5,11,12,14,15^

## Learning points

1. Spinal cord intramedullary lipomas are rare, comprising 2% of intramedullary tumours.
2. They are more often associated with spinal dysraphism.
3. On MRI these lesions show typical signal characteristics of fat.
4. They are benign; however complete surgical removal is usually impossible due to extensive attachment to the surrounding neural tissues.
5. Massive spinal cord intramedullary lipomas are uncommon.
6. Bilocular spinal cord intramedullary lipomas have not been reported in the literature.

## Consent

Written informed consent for the case to be published (including images, case history and data) was obtained from the patient(s) for publication of this case report, including accompanying images.
